# Lifestyles and academic stress among health sciences students at the National University of Chimborazo, Ecuador: a longitudinal study

**DOI:** 10.3389/fpubh.2024.1447649

**Published:** 2024-08-12

**Authors:** Yolanda E. Salazar-Granizo, César Hueso-Montoro, Rafael A. Caparros-Gonzalez

**Affiliations:** ^1^Doctorate Program in Clinical Medicine and Public Health, University of Granada, Granada, Spain; ^2^Instituto de Investigación Biosanitaria (ibs-GRANADA), Granada, Spain; ^3^Department of Nursing, Faculty of Health Sciences, National University of Chimborazo, Riobamba, Ecuador; ^4^Department of Nursing, Faculty of Health Sciences, University of Jaén, Jaén, Spain; ^5^Center for Mind, Brain, and Behavior Research (CIMCYC), Granada, Spain; ^6^Department of Nursing, Faculty of Health Sciences, University of Granada, Granada, Spain

**Keywords:** stress, lifestyle, online education, social isolation, health students

## Abstract

**Background:**

The significant changes experienced by university students in their training are inherent to educational processes. Social isolation caused by the COVID-19 pandemic, online education and the reopening of higher-education institutions produced substantial variations in the lifestyle of university students in health sciences and generated academic stress and perceived stress. This study was conducted at the National University of Chimborazo (UNACH), a public institution located in Riobamba, central Ecuador, the diverse student's population provided an ideal setting for examining the interplay between academic and perceived stress, lifestyle factors, and learning modalities. The research focused on health sciences students across six academic programs. The university's geographical position and demographic characteristics offered a representative sample for investigating these factors within the context changing.

**Aim:**

To compare academic and perceived stress and university students' lifestyles at two different periods: (1) during the mandatory social confinement caused by the COVID-19 pandemic with an online learning modality (T1); (2) in the post-pandemic period with a return to face-to-face activities (T2).

**Design:**

An observational, analytical, quantitative, and longitudinal study.

**Participants:**

Students from six programs (Nursing, Physiotherapy, Clinical Laboratory, Medicine, Dentistry, and Clinical Psychology) from the Faculty of Health of the National University of Chimborazo-Ecuador (*n* = 2,237) participated voluntarily, the students had one mean age of M = 21.31 (SD = 2.36) at T1 and M = 22.94 (SD = 2.40) at T2. Non-probability convenience sampling was employed due to the accessibility of the student population and the importance of including the maximum number of relevant individuals within the study population.

**Methods:**

The following instruments were used: Nola Pender's Lifestyle Profile Questionnaire, Cognitive Systemic Inventory for the study of academic stress, and Cohen's Perceived Stress Scale.

**Results:**

In T1 and T2, students reported high levels of stress, and increased unhealthy lifestyle increased with the return to classrooms. Additionally, upon returning to face-to-face activities (T2), the mean score applied to the responses of Nola Pender's Lifestyle Profile Questionnaire decreased from M = 113.34 (SD = 23.02) to M = 107.2 (SD = 29.70; *p* < 0.001). There was significant difference (*p* < 0.001) in academic stress in T1 [M = 66.25 (SD = 15.66)] and T2 [M = 64.00 (SD = 17.91)].

**Conclusions:**

Upon returning to university classrooms (T2), the number of students who reported an “unhealthy” lifestyle increased. Academic stress was high in T1 and T2 and was reported higher in online activities during social isolation.

## 1 Introduction

University students experience significant changes during their training, such as an increase in autonomy, greater responsibility, and life stressors, which affect their mental health, wellbeing, and commitment to their health (1, 2). Lifestyle, defined as conditions, behaviors, and habits chosen by individuals (3), constitutes an indicator of wellbeing, higher productivity, and life expectancy (4). Stressful factors are poor behaviors and habits chosen by individuals as part of their lifestyles (5).

The World Health Organization declared COVID-19 global pandemic on the March 11, 2020, after outbreaks were reported in more than 110 countries (6). This global emergency was considered one of the greatest economic and health disasters in history (7, 8). The owing to social isolation substantial changes occurred in the lifestyle of university students in health sciences. Further, high levels of academic stress (5), were generated by, among other factors, online teaching, and instruction (9). University students' lifestyles were modified during social isolation. These modifications affected their physical activities, generated sedentary behaviors, and caused changes in diet and sleep routines (10–13).

Stress is an individual's adaptive response to cope with adverse events. However, when it extends over time, it is considered one of the greatest toxins for health (14, 15). This systemic adaptive process generated by students in teaching and learning environments is known as academic stress (16). Academic stress is related to psychosomatic symptoms, such as sweating, tremors, exhaustion, and irritable bowel (17), and potentially causes depression, poor academic performance, and even school dropout (18).

Social isolation generated by adverse events, such as the pandemic, impacted university teaching, especially causing a transition from face-to-face learning to online education (19, 20). A previous study reported that problems of online education, such as difficulties with internet connectivity; poor quality or lack of technological equipment; eye strain; fatigue; and less interaction with peers, teachers, and health personnel diminished learning efficiency, which eventually, in the case of health of students, affected their morale, mental wellbeing, and self-confidence (21). Mandatory social isolation measures imposed by health authorities led higher-education institutions to abruptly transition from face-to-face teaching to online education modalities (19, 20).

The return to in person activities, mainly due to mass vaccination, often termed “returning to normalcy,” and the pressure to recover time lost during the pandemic have compelled populations to contend with elevated levels of anxiety, depression, and stress (22, 23). The pandemic unveiled persistent social inequalities that have endured beyond the critical phase. Public policies supporting mental health remain inadequately established, highlighting a lack of empathy toward the challenging post-pandemic reality (24). This situation underscores the need for comprehensive mental health interventions and policy reforms to address the long-term psychological impacts of the pandemic COVID-19. In Ecuador, 13.6 million people received the vaccine against COVID-19 as of March 14, 2022 (25) so the total of face-to-face activities were resumed from this date.

The reopening of higher education institutions posed new challenges to training. The return to face-to-face education required universities to implement safety protocols and maintain social and physical distancing to minimize the risk of infection (26, 27). Additionally, new strategies had to be adopted, and curricula had to be reconfigured and reconsidered to strengthen virtually imparted clinical skills (28). These adaptations aimed to promote experiential learning (encompassing concrete experience, reflective observation, abstract conceptualization, and active experimentation) (29) foster active student participation, and contribute to the acquisition of practical learning outcomes, and introduce modifications to students' lifestyles and analyze the stress levels (30).

The National University of Chimborazo is a public higher education institution established in August 1995 in Riobamba, Ecuador (31). Located in the country's central region within administrative zone three, UNACH's strategic geographic position, favorable climate, and the city's affordable cost of living attract students from diverse regions across Ecuador. The university offers programs in Engineering, Education, Administration, and Health Sciences, with the latter comprising six distinct academic programs (25).

In response to the global COVID-19 pandemic, UNACH suspended in-person activities on March 17, 2020, transitioning to online instruction, this shift particularly impacted the Faculty of Health Sciences, due to the necessitating the suspension of formation hospital-based and simulation laboratory's practices (32). Following the implementation of government-led mass vaccination initiatives and institutional measures for a gradual return to the university campus, UNACH announced the resumption of activities on September 28, 2020, with a proportional (30%) of the administrative staff (33). The full return to on-campus activities for the entire university community (students, faculty, administrative staff, and workers) was established on November 7, 2022 (34).

The university's diverse student population and its strategic importance within Ecuador's higher education system provided an ideal setting for examining the interplay between academic stress, lifestyle factors, and learning modalities among health sciences students. This study uniquely captures these dynamics across two distinctly different educational, social, economic, and familial contexts: the period online learning during pandemic restrictions and the subsequent return to in-person instruction.

In this context, the present longitudinal study aimed to compare academic stress and lifestyles in university students of health sciences in two different contexts: during mandatory social confinement when online learning was a modality and during the students' return to face-to-face activities. The following research question was posed: Varies the lifestyle patterns and academic stress levels among health sciences university students, in different educational contexts?

## 2 Methods

### 2.1 Design, study population, and procedure

An observational, analytical, and longitudinal study with a repeated-measures design was conducted. The study population comprised students (*N* = 2,880) enrolled in the Nursing, Physiotherapy, Clinical Laboratory, Medicine, Dentistry, and Clinical Psychology programs at the Faculty of Health Sciences of the National University of Chimborazo (Ecuador).

All enrolled students were invited to participate voluntarily in this longitudinal study, which spanned two distinct academic periods: the final online learning period due to mandatory social isolation (T1), and the first period of in-person learning upon return to university classrooms (T2). Non-probability convenience sampling was employed due to the accessibility of the student population and the importance of including the maximum number of relevant individuals within the study population and know his appreciation regarding these two moments of change.

Inclusion criteria were: (1) enrollment in the institution at the time of the application of the questionnaires, (2) being over 18 years of age, and (3) completing all data collection instruments in both periods (T1 and T2), which made it possible to ensure the consistency of the data in the two moments. Due to pandemic-related restrictions and mandatory social distancing during both the COVID-19 and post-COVID-19 phases, a virtual approach was implemented to invite student participation. An invitation email, accompanied by the participant information document, was sent to students. Additionally, synchronous virtual activities were arranged via online platforms to communicate the project's objectives and scope, emphasize voluntary participation, and encourage students to complete the questionnaires. With necessary approvals from the university and faculty, data were collected online through the University's Academic System (SICOA). This approach resulted in active student participation in this study.

**Figure 1:**
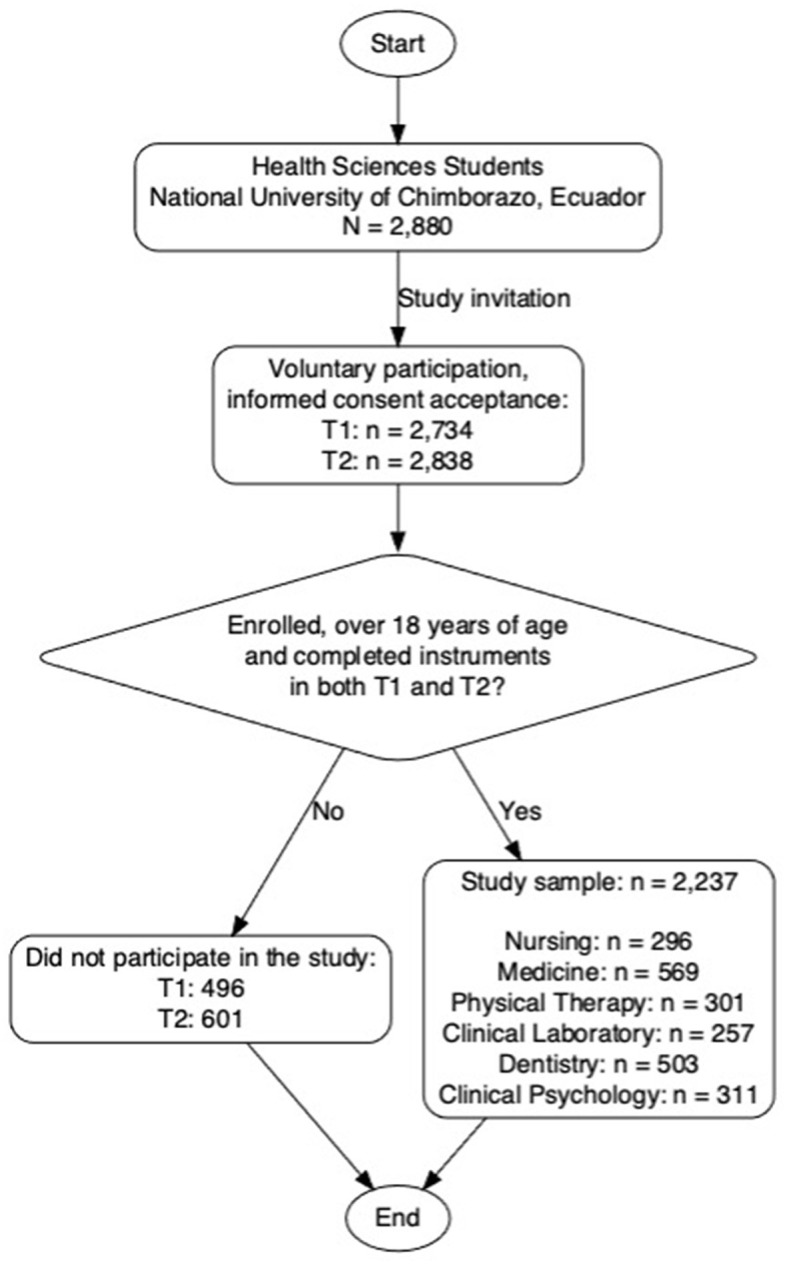
Student engagement flow. T1 academic period characterized by the online-learning modality owing to the mandatory social isolation imposed by the COVID-19 pandemic. T2 academic period, in which students returned to face-to-face activities.

The participation rate was high in both phases: 94.93% (*n* = 2,734) in T1 and 98.54% (*n* = 2,838) in T2. For the longitudinal analysis, data from 2,237 students who met all criteria were included, providing a high representation of the eligible student population of the academic programs Nursing (*n* = 296); Medicine (*n* = 569); Physical Therapy (*n* = 301); Clinical Laboratory (*n* = 257); Dentistry (*n* = 503); Clinical Psychology (*n* = 311), of these, 71.1% (*n* = 1,591) were women. The vast majority were single, with proportions of 96.8% (*n* = 2,165) in T1 and 97.5% (*n* = 2,180) in T2. The mean age of the students was 21.31 years (SD = 2.36) in T1 and 22.94 years (SD = 2.40) in T2. This significant sample allowed for a holistic view of the subject matter across both time points (Figure 1).

### 2.2 Data collection phases

The research was developed in two data-collection phases with two differentiated-measurement points:

Initial Phase (T1): 2022-1S academic period (April 18, 2022, to August 4, 2022), characterized by the online-learning modality owing to the mandatory social isolation imposed by the COVID-19 pandemic.

**Figure 2:**
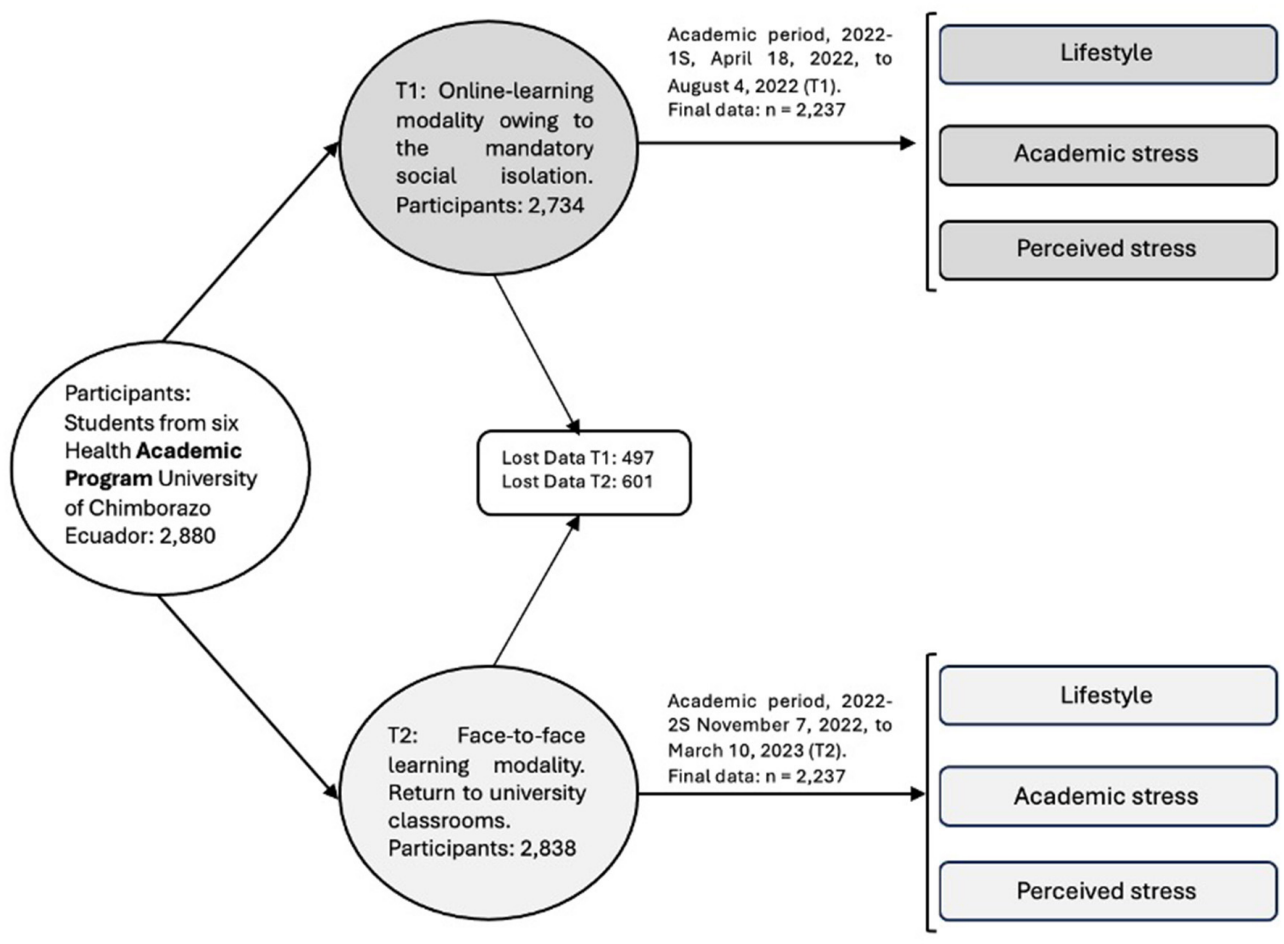
Longitudinal design, study sample, and procedure.

Phase 2 (T2): 2022-2S academic period (November 7, 2022, to March 10, 2023), in which students returned to face-to-face activities. The participation rate was 94.93% (*n* = 2,734) in T1 and 98.54% (*n* = 2,838) in T2 (Figure 2).

### 2.3 Ethical considerations

The present study follows fundamental principles outlined in the Declaration of Helsinki fundamental ethical principles emanating from the Declaration of Helsinki. The research protocol was evaluated and approved by a Human Research Ethics Committee approved by the Ministry of Public Health of Ecuador.

To safeguard the confidentiality of the data provided by the participants, procedures for anonymizing the collected information were implemented, and the possibility of individual identification of the students was minimized. Prior to the start of the study, informed consent was obtained from all participants, who were duly informed about the objectives, methodologies, risks, and benefits of the research.

Throughout all phases of the research process, the bioethical principles of non-maleficence, beneficence, autonomy, and justice were observed, and compliance with the ethical norms and standards required in scientific research involving human beings was thus ensured.

### 2.4 Instruments

Changes in lifestyle dimensions, academic stress, and perceived stress were assessed through three scales previously validated in the Ecuadorian population. Data, along with demographic information from participants in the two study phases, were collected online on the National University of Chimborazo's academic platform (T1 and T2).

To quantitatively measure the level of lifestyle, Nola Pender's Lifestyle Profile Questionnaire was utilized. This instrument evaluates six dimensions: nutrition, exercise, health responsibility, stress management, interpersonal support, and self-actualization, through 48 items. Higher scores, ranging from 48 to 192 points, indicate a healthier lifestyle. The scale validated by Habibzadeh et al. (35) reports a Cronbach's alpha of 0.94 for the entire questionnaire.

The Cognitive Systemic Inventory for the Study of Academic Stress, developed by Barraza, was employed to assess academic stress; physical, psychological, and behavioral symptomatology; and coping strategies. This instrument consists of 21 items, and its results are established in a range from 21 to 105 points, where higher scores indicate greater academic stress. In a previous study, Ruiz Camacho and Barraza-Macías (36) reported an internal reliability using Cronbach's alpha of 0.85. To measure perceived stress and coping, Cohen et al.'s (37) Perceived Stress Scale was used. This scale consists of 14 items that are used to explore the feelings and thoughts of stress that individuals have experienced for the last months. Scores range from 0 to 56 points, with higher values indicating greater perceived stress. Larzabal-Fernandez et al. (38) validated this instrument, establishing an internal reliability using Cronbach's alpha of 0.80.

Additionally, demographic information was collected, such as age (distributed into four age groups), sex, marital status (single, married, divorced, and cohabiting), economic dependence (parents, relatives, partner, or other), health career, level of study (from 1st year to rotating internship, classified according to current national higher education regulations), academic average (on a scale from zero to 10), and participants' nationality.

### 2.5 Data analysis

Initially, a descriptive approach was used for statistical analysis to summarize the sociodemographic characteristics and other variables of the participating students. Frequencies and percentages were calculated for categorical variables, while means and standard deviations were reported for continuous variables.

To evaluate differences between two phases of the study (T1 and T2), various statistical techniques were employed. Association tests based on the chi-square statistic (χ^2^) and Student's *t*-test for paired samples were used to compare means. Furthermore, a repeated measures analysis of variance (ANOVA) was conducted to compare the study population in the two analyzed contexts.

All analyses were performed using the Statistical Package for Social Sciences (SPSS), version 24.0. A statistical significance level of *p* < 0.05 was established for the interpretation of the results.

## 3 Results

### 3.1 Demographic data

Ecuador, is a South American nation, divided into 24 provinces and nine administrative planning zones, boasted a total population of 17,629,765; of them 2,175,000 were the university-age bracket of 18–24 years in 2022 (39). The nation's constitution mandates free undergraduate education at public universities (40).

National University of Chimborazo, a public institution, attended the educational needs of a high number student through 31 undergraduate and 23 graduate programs during research period, the institution is situated in Riobamba, Chimborazo province, in central Ecuador and is a reference institution for higher education in the region (41).

During the T1 research period, UNACH's undergraduate programs and pre-professionals' internships encompassed 10,252 students. Of this cohort, 58.72% (*n* = 6,020) were female, 46.88% (*n* = 4,807) hailed from 23 provinces outside the institution's locale. In the T2 period, the total student population decreased to 10,123. Male students constituted 40.84% (*n* = 4,135) of this group, while 53.6% (*n* = 5,426) originated from Chimborazo Province, where the institution is located (41).

The Faculty of Health Sciences housed of the 28.08% of students in T1 and 28.25% in T2. From this pool, 2,237 students met the inclusion criteria, of them the 71.1% percent (*n* = 1,591) were women. The vast majority were single, with proportions of 96.8% (*n* = 2,165) in T1 and 97.5% (*n* = 2,180) in T2. The mean age of the students was 21.31 (SD = 2.36) years in T1 and 22.94 (SD = 2.40) years in T2. Regarding financial dependence, a substantial decrease was observed between both phases, with the percentage of students economically dependent on their parents dropping from 90.7% (*n* = 2,028) in T1 to 77.6% (*n* = 1,735) in T2.

Academic performance showed a decrease in the proportion of students with grades in the excellent range (9–10 from 17.38% (*n* = 389) in T1 to 9.87% (*n* = 221) in T2. Consequently, the average grade also decreased from 8.27 (SD = 0.71) in T1 to 8.10 (SD = 0.92) in T2 (Table 1).

**Table 1:** Demographic data of university students during T1 and T2 (*n* = 2,237).

**Variables**	**First time point (T1)**			**Second time point (T2)**		
**Age** ^*^	**Frequency**	**%**	**M (SD)**	**Frequency**	**%**	**M (SD)**
18–24	1,823	81.5	21.31 (2.36)	2,076	92.8	22.94 (2.40)
25–31	397	17.7		152	6.8	
32–38	13	0.6		7	0.3	
39+	4	0.2		2	0.1	
**Sex**						
Woman	1,591	71.1		1,591	71.1	
Man	646	28.9		646	28.9	
**Marital status**						
Single	2,165	96.8		2,180	97.5	
Married	52	2.3		31	1.4	
Cohabiting	7	0.3		3	0.1	
Divorced	13	0.6		23	1	
**Financial dependence**						
Not applicable	81	3.6		439	19.6	
Parents	2,028	90.7		1,735	77.6	
Family	88	3.9		48	2.1	
Couple	23	1		14	0.6	
Other	17	0.8		1	0	
**Academic program**						
Nursing	296	13.20		296	13.20	
Medicine	569	25.40		569	25.40	
Physical therapy	301	13.50		301	13.50	
Clinical laboratory	257	11.50		257	11.50	
Dentistry	503	22.50		503	22.50	
Clinical psychology	311	13.90		311	13.90	
**Level**						
First	491	21.90		311	13.90	
Second	215	9.60		220	9.83	
Third	279	12.50		222	9.92	
Fourth	352	15.70		287	12.83	
Fifth	262	11.70		330	14.75	
Sixth	271	12.10		276	12.34	
Seventh	126	5.60		233	10.42	
Eighth	149	6.70		154	6.88	
Ninth	37	1.70		118	5.27	
Tenth	42	1.90		72	3.22	
Internship rotation	13	0.58		14	0.63	
**Grade point average** ^*^						
Excellent (9–10)	389	17	8.27 (0.71)	221	9.8	8.10 (0.92)
Very good (8–8.9)	1,151	51.4		1,172	52.3	
Good (7–7.9)	625	27.9		694	31.0	
Fail (< 7)	72	3.2		150	6.7	

^*^The variables are presented in the table in classes with intervals to facilitate visualization. However, the statistical analyses were performed using the original data collected as discrete quantitative variable (age) and continuous quantitative variable (grade point average).

%, percentage; M, mean; SD, standard deviation.

### 3.2 Findings on lifestyles, academic, and perceived stress

In both phases of the study, students reported a moderately healthy lifestyle. However, a significant decrease in the means of the six dimensions on the Nola Pender Lifestyle Profile scale was evident during the T2 phase compared to T1. For instance, the “health responsibility” dimension showed a decrease in the mean from 19.85 (SD = 5.51) in T1 to 18.14 (SD = 8.10) in T2 (*p* < 0.001).

**Figure 3:**
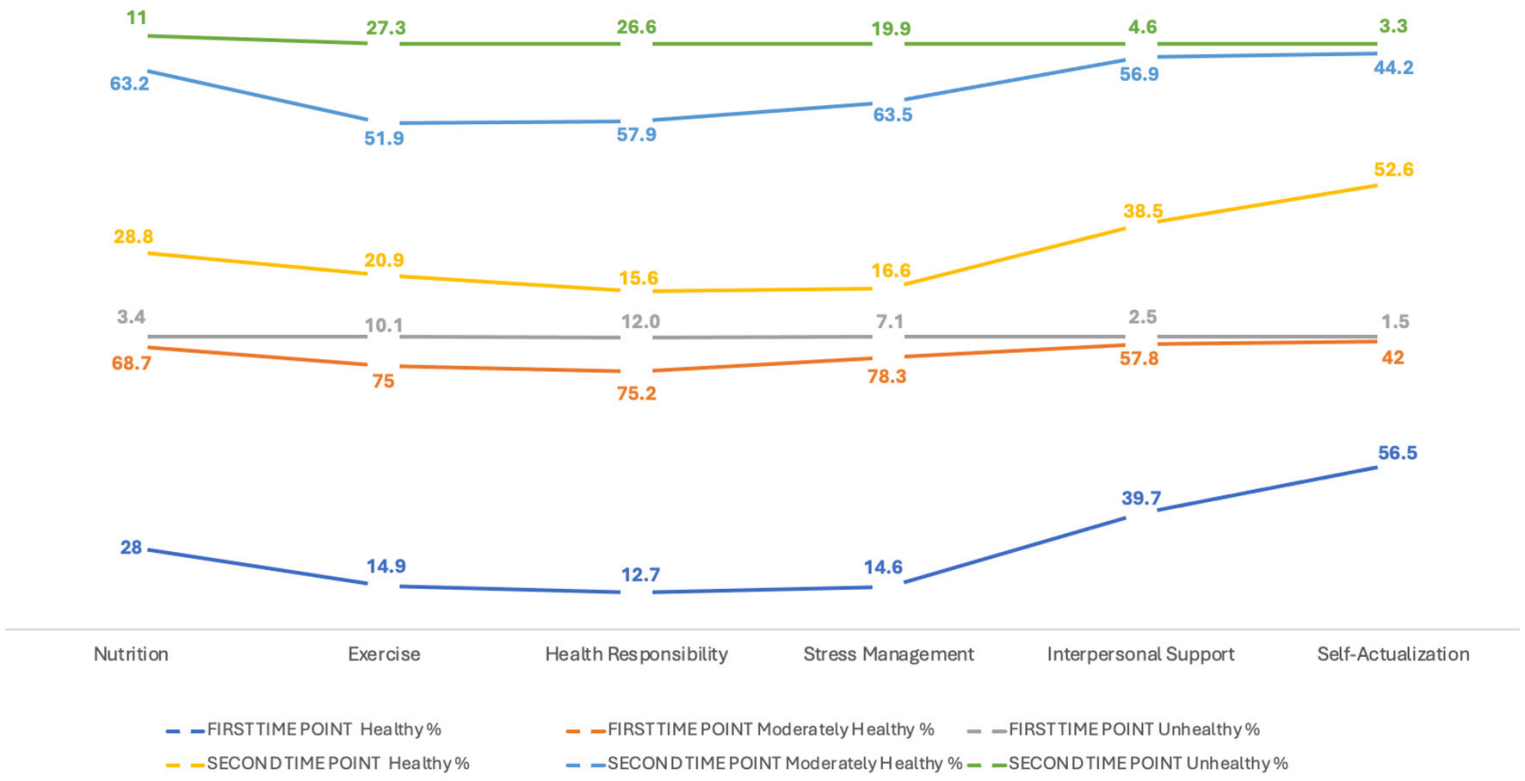
Lifestyle dimensions T1–T2 (*n* = 2,237).

Associated with the above, an increase in the frequency of students who reported an unhealthy lifestyle in T2 was observed. The most affected dimensions were “exercise,” with 27.3% (*n* = 610) of students in the unhealthy range in T2 compared to 10.1% (*n* = 225) in T1, and “health responsibility,” with 26.6% (*n* = 594) in T2 compared to 12% (*n* = 269) in T1 (Figure 3).

**Figure 4:**
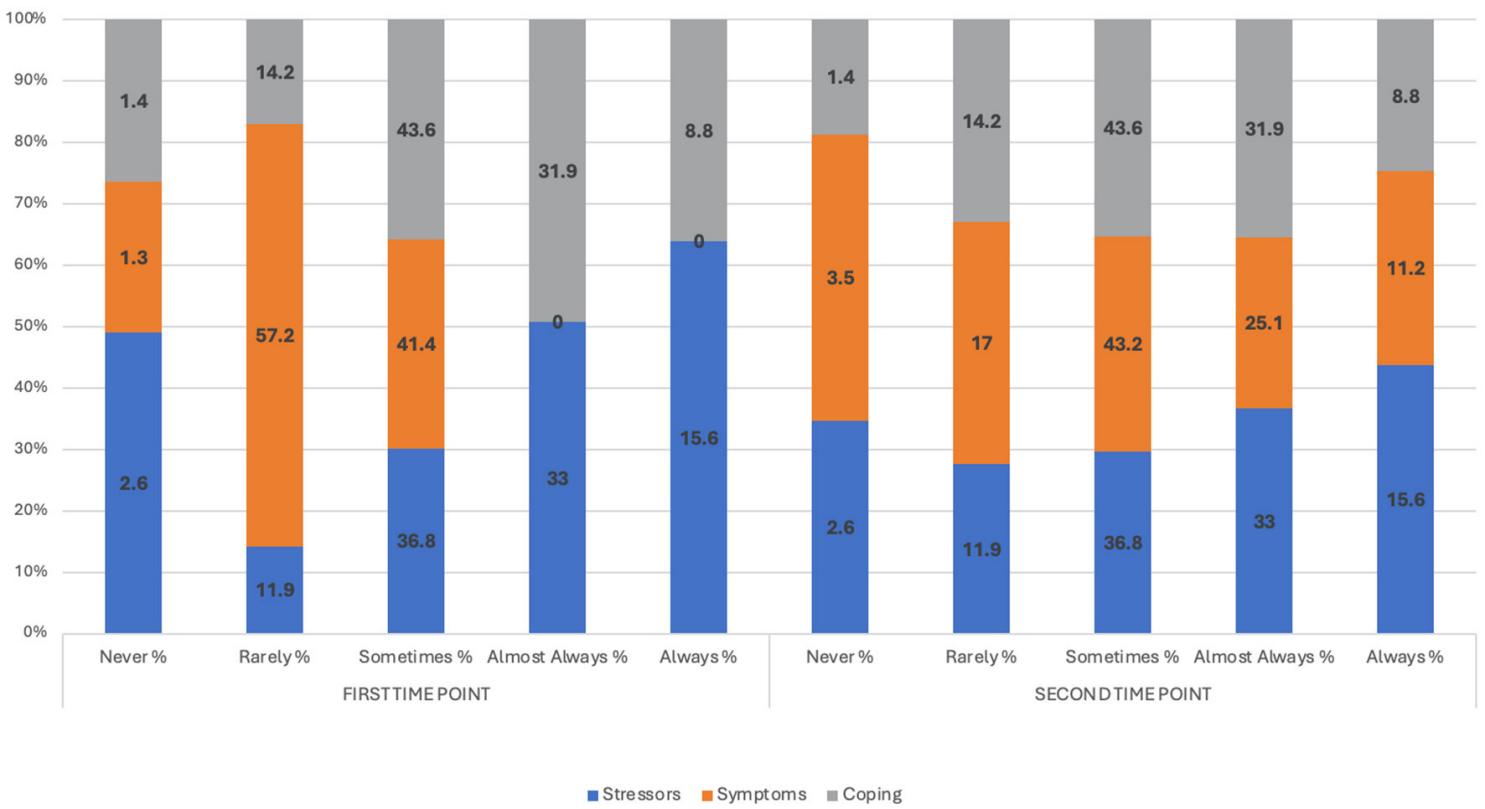
Academic stress dimensions T1–T2 (*n* = 2,237).

Students reported having experienced academic stress in T1. In T2, the means in the three dimensions of the scale slightly decreased. However, 25.1% (*n* = 562) of students indicated having presented stress symptoms almost always or 11.2% (*n* = 251) of students indicated it in T2 in categories not manifested in T1 (Figure 4).

In the chi-square statistical analysis conducted in the two phases of the longitudinal study, a significant relationship was found between the students' lifestyle and the sex variable (*p* = 0.001). Men presented a higher percentage of healthy lifestyles (32.4%; *n* = 209) compared to women (22.9%; *n* = 364) in both phases of the study.

Additionally, a significant difference in healthy lifestyles was observed among the various health sciences programs (*p* = 0.005). Students in the medicine (29.3%; *n* = 167) and nursing (29.7%; *n* = 88) programs showed the highest percentages of healthy lifestyles, while those in the dentistry (22.3%; *n* = 112) and clinical laboratory (20.2%; *n* = 52) programs presented the lowest percentages (Supplementary material 1).

Academic stress was significantly associated with marital status (*p* < 0.001) in T1. Single students presented moderate academic stress more frequently (62.3%; *n* = 1,358) than students who were married, cohabiting, and divorced. Additionally, students who financially relied on their parents presented moderate academic stress in 62.8% (*n* = 1,272) of cases, which also showed a significant association (*p* < 0.001).

Women reported severe academic stress more frequently in both T1 (38.6%; *n* = 613) and T2 (33.3%; *n* = 530; *p* = 0.007).

Students doing dentistry (41.6%; *n* = 209) and clinical psychology (38.3%; *n* = 119) programs presented severe academic stress more frequently in T1 (*p* = 0.005), while in T2, those doing dentistry (39.2%; *n* = 197) and medicine (32.9%; *n* = 187) showed a lower frequency of severe academic stress (*p* = 0.003; Supplementary material 2).

Perceived stress was presented similarly in the same variables during the two phases of the study. In both phases, women most frequently perceived stress “occasionally,” representing 44.7% (*n* = 711) in T1 and 47.5% (*n* = 755) in T2.

Likewise, students in the fifth academic level most frequently perceived stress, reaching 53.4% (*n* = 140) in T1 and 50.6% (*n* = 167) in T2. Moreover, students who financially relied on their partners or relatives more frequently perceived stress, being 47.8% (*n* = 11) in T1 and 50% (*n* = 24) in T2 (Supplementary material 3).

Also, women were found to “occasionally” use stress-coping strategies more frequently, representing 52% (*n* = 827) in T2 of the study. Similarly, single students “occasionally” resorted to stress-coping strategies more frequently, reaching 51.7% (*n* = 1,126) in T1. This relationship between marital status and stress-coping strategies was statistically significant (*p* = 0.001; Supplementary material 4).

Students showed a decrease in the means of their healthy lifestyles in the two phases of the longitudinal study. The mean on the lifestyle scale was 113.34 (SD = 23.02) during T1 and decreased to 107.2 (SD = 29.70) in T2. This difference was statistically significant (*t* = 10.49, *p* < 0.001), with a moderate effect size of 0.47. These findings suggest that the students' lifestyle worsened after they returned to face-to-face activities compared to virtual activities.

Regarding academic-stress levels, the results showed a significant decrease in what was reported by students throughout the two phases. The mean scores on the academic stress scale were 66.25 (SD = 15.66) in T1 and decreased to 64.00 (SD = 17.91) in T2. This difference was statistically significant (*t* = 5.48, *p* < 0.001), with a moderate effect size of 0.34. These findings suggest that academic stress decreased after students returned to face-to-face activities.

Perceived stress and coping with it decreased upon students' return to face-to-face activities. For perceived stress, in T1 M = 13.28 (SD = 4.82) and in T2, M = 12.85 (SD = 4.94; *t* = 3.70, *p* < 0.001), with an effect size of 0.37. For coping with stress, in T1, M = 21.54 (SD = 5.69) and in T2, M = 21.02 (SD = 6.40; *t* = 3.27, *p* = 0.001), with an effect size of 0.25. The results show a significant decrease in perceived stress and coping with it in the phase two of the study.

Additionally, a significant decrease in the levels of perceived stress and stress coping was reported by the students in the two phases. For perceived stress, the mean was 13.28 (SD = 4.82) in T1, which decreased to 12.85 (SD = 4.94) in T2 (*t* = 3.70, *p* < 0.001), with a moderate effect size of 0.37. For stress coping, the mean was 21.54 (SD = 5.69) in T1 which decreased to 21.02 (SD = 6.40) in T2 (*t* = 3.27, *p* = 0.001), with a small effect size of 0.25. These results indicate that both perceived stress and stress coping decreased significantly in the phase two of the study (see Table 2).

**Table 2:** *T*-test, lifestyles, perceived stress, and academic stress (*n* = 2,237).

**Variables**	**M (SD)**	***t* (*p*)**	**Effect size**
**Lifestyles**			
T1	113.34 (23.02)	10.49 (0.001)	0.47
T2	107.2 (29.70)		
**Academic stress**			
T1	66.25 (15.66)	5.48 (0.001)	0.34
T2	64.00 (17.91)		
**Perceived stress**			
T1	13.28 (4.82)	3.70 (0.001)	0.37
T2	12.85 (4.94)		
**Perceived coping**			
T1	21.54 (5.69)	3.27 (0.001)	0.25
T2	21.02 (6.40)		

M, mean; SD, standard deviation; t, calculated t-value; (p), p-value (statistical significance).

## 4 Discussion

The present longitudinal study aimed to compare the lifestyles, academic stress, and perceived stress of the students of health sciences at the National University of Chimborazo-Ecuador related to the online-learning mode during the mandatory isolation period and after their return to face-to-face activities.

### 4.1 Main findings

On the return in-person activities, a decrease in lifestyle dimensions was observed among students, compared to the values reported during online learning resulting from mandatory social isolation. Males they had better lifestyle in both phases of the study. Students in Medicine and Nursing programs showed the highest percentages of healthy lifestyles, while those in Dentistry and Clinical Laboratory programs presented the lowest percentages.

Levels of academic stress decreased upon returning to in-person activities compared to the virtual learning stage; females reported higher levels of severe academic stress and perceived stress than males in both stages of the study. Students in Dentistry and Clinical Psychology programs exhibited higher severe academic stress in T1; the students in dentistry and Medicine programs showed a lower frequency of severe academic stress in T2.

Perceived stress and coping with it decreased upon students' return to face-to-face activities. In both phases, women most frequently perceived and faced stress “occasionally.”

### 4.2 Comparison with previous studies

This study was conducted with a sample of 2,237 students from six academic programs health, with a prevalence of female students, that which is consistent with research on lifestyles and academic stress in university students (42–50).

The findings revealed a significant change in students' healthy lifestyles after they returned to face-to-face activities compared to the online learning stage during isolation. Specifically, the dimensions of exercise and health responsibility were the least observed factors of health at both time points. This result is consistent with previous studies in Ecuador, that they mentioned the low physical activity levels among university students (51), as well as the increased daily time spent sitting in front of technological devices, with a predominance in the female gender during mandatory social isolation (42). In the post-pandemic stage, results show the prevalence of physical inactivity, especially among male students in medical programs (43).

Research on university students in America and Europe have reported that activities affecting lifestyle, such as the perception of inadequate nutrition and difficulties in sports and social activities, were concomitant with social isolation (44, 52). However, these findings differ from those reported by Tárraga Marcos et al. (45), after confinement, who found an improvement in participants' adherence to healthy diets and physical activity. These differences could be due to academic and cultural particularities of the study contexts.

Regarding academic stress, the results showed a significant decrease in stress levels, symptoms, and coping strategies reported by students upon returning to face-to-face activities compared to the virtual-learning stage. These findings contrast with previous research that has documented an increase in stress, anxiety, depression, and other negative symptoms associated with the distance-learning modality (46–48) and the progressive decrease of these in the post-pandemic stage (53). Students' preference for traditional face-to-face education and the perception of “ineffective teaching” during virtual learning (8, 54–56) could explain the decrease in academic stress when the students return to face-to-face activities.

Notably, at both time points, women reported higher levels of severe academic stress and perceived stress than men. These results are consistent with those of previous research indicated that women presented higher levels of sadness, stress, anxiety, and depression with a stronger psychological impact (9, 49, 57, 58). In additionally, Reivan et al. report that poorly regulated emotions and being female increase the severity of perceived stress in university students. This result is consistent across all Ecuadorian regions (Insular, Coast, Highlands, and Amazon) (50).

A notable finding of the present study was the increase in the number of students who failed when they returned to face-to-face activities. This could be due, in part, to the cognitive deterioration experienced by those students who were infected with COVID-19 during the isolation period (59). Likewise, the change in learning modality, especially regarding the practical components of health sciences programs, could have represented a significant challenge for students (60). The transition from a virtual learning environment to a face-to-face one, with the demands and requirements inherent to field and laboratory activities, could have negatively affected the participants' academic performance. The university students In Ecuador, expressed the negative impact of the pandemic on health education and mentioned concern about the lack of improvement upon returning to in-person education, as the learning strategies remain the same as those used prior to the pandemic (61).

The measurement of the scale Nola Pender's Lifestyle Profile and the Cognitive Systemic Inventory for the Study of Academic Stress generally showed a decrease in the mean among health students at the University of Chimborazo upon returning to face-to-face activities compared to these same variables in virtual activities. These results differ from those reported by Berdida and Grande (21), who found a significant negative relationship between academic stress and the lifestyle and quality of life of health students—as academic stress increases, students' quality of life decreases.

### 4.3 Limitation and recommendations for future research

Replicating the longitudinal study in diverse samples, with health sciences students from different universities and regions, both nationally and internationally, will allow for evaluating whether observed patterns in lifestyle, academic stress, and perceived stress are maintained or vary depending on different sociocultural and academic environments. Additionally, including possible factors that influence the experience of transitions between teaching modalities, such as health and academic policies and student support systems, could impact the results.

### 4.4 Strengths of the study

The greatest relevance of this longitudinal study lies in the large sample size of participating students, which provides robustness to the reported results. Furthermore, the data collection at two key moments—during the mandatory social isolation with virtual learning modality and upon returning to face-to-face activities—represents another methodological strength as it captures students' perceptions in real-time, avoiding recall bias. This gives a more accurate picture about lifestyle changes and the generation of academic stress throughout transitions between teaching modalities.

### 4.5 Implications for educational practice

The completion of the stage of drastic experiences in higher education owing to the COVID-19 pandemic requires reflecting on multiple changes that have impacted students' lives. Understanding these impacts on students' lifestyles and the generation of stress in response to extreme contexts is fundamental to facing future interruptions in academic scheduling can help prevent such disruptions from negatively affecting the quality of education.

In this sense, the incorporation of technological tools and innovative resources into education, especially in the field of clinical practice, can be considered a valuable complement. These elements can facilitate the delivery of content, strengthen skills, and support the appropriation of learning outcomes and competencies in the training of a new generation of health professionals. However, these innovative strategies should not replace face-to-face training activities, which remain a fundamental component in higher education in the health sciences.

## 5 Conclusions

Health-science students at the National University of Chimborazo maintained moderately healthy lifestyles both during the mandatory social isolation and upon returning to face-to-face activities in university classrooms. However, an increase in the percentage of students who reported “unhealthy lifestyles” was observed upon the students' return to face-to-face activities, especially in the dimensions of exercise and health responsibility. This is probably because multiple activities academic was performed outside the home and the time dedicated to healthy lifestyles decreased.

Also, students experienced academic stress at both time points of the study. However, a significant decrease in the dimensions of stressors, symptoms, and coping strategies was found upon the students' return to face-to-face activities, compared to the online learning stage. The levels of perceived stress and coping did not show relevant changes between the two time points.

The results also indicate that men and students in the Medicine and Nursing programs reported healthier lifestyles. Furthermore, women presented severe academic stress more frequently, although this type of stress decreased slightly upon their return to face-to-face activities. Dentistry students, at both time points, reported the highest levels of severe academic stress.

## Data availability statement

The raw data supporting the conclusions of this article will be made available by the authors, without undue reservation.

## Ethics statement

The studies involving humans were approved by Ethics Committee Universidad Católica de Cuenca; Ministry of Public Health of Ecuador. The studies were conducted in accordance with the local legislation and institutional requirements. The participants provided their written informed consent to participate in this study.

## Author contributions

YS-G: Conceptualization, Data curation, Formal analysis, Investigation, Methodology, Writing – original draft, Validation. CH-M: Supervision, Visualization, Writing – review & editing. RC-G: Supervision, Visualization, Writing – review & editing.
