# Supplementary material for: Lifestyles and academic stress among health sciences students at the National University of Chimborazo, Ecuador: a longitudinal study
Source: Front Public Health. 2024 Aug 12;12:1447649. doi: 10.3389/fpubh.2024.1447649 (PMC11345227; doi:10.3389/fpubh.2024.1447649)
Supplement: Supplementary file 1 [file Table_1.pdf]

Supplementary material 1*: Nola Pender Lifestyles Association X^2 (n= 2237) T1 y T2.*

Variables	First Moment (T1) Unhealthy	Moderately Healthy	Healthy	p T1	Second Moment (T2) Unhealthy	Moderately Healthy	Healthy	p T2
	fi. (%)	fi. (%)	fi. (%)		fi. (%)	fi. (%)	fi. (%)	
**Sex**								
Man	11 (1.7)	426 (65.9)	209 (32.4)	0.001	11 (1.7)	426 (65.9)	209 (32.4)	0.001
Woman	24 (1.5)	1,201 (75.5)	364 (22.9)		26 (1.63)	1,201 (75.4)	364 (22.8)	
**Age**								
18 - 24	32 (1.5)	1,279 (61.6)	764 (36.8)	0.001	57 (3.1)	1,166 (64.0)	600 (32.9)	0.001
25 - 31	5 (3.3)	112 (73.7)	35 (23.09		16 (4.0)	275 (69.3)	106 (26.7)	
32 - 38	1 (14.3)	3 (42.9)	3 (42.9)		0 (0.0)	7 (53.8)	6 (46.2)	
39+	0 (0.0)	2 (100.0)	0 (0.0)		2 (50.0)	1 (25.0)	1 (25.0)	
**Marital status**								
Single	34 (1.6)	1,591 (73)	555 (25.5)	0.11	33 (1.5)	1,577 (72.8)	555 (25.6)	0.002
Married	3 (9.7)	21 (67.7)	7 (22.6)		4 (7.7)	40 (76.9)	8 (15.4)	
Divorced	0 (0.0)	2 (66.7)	1 (33.3)		0 (0.0)	4 (57.1)	3 (42.9)	
Cohabiting	0 (0.0)	13 (56.5.)	10 (43.5)		0 (0.0)	6 (46.2)	7 (53.8)	
**Financial Dependence**								
Not applicable	3 (3.7)	49 (60.5)	29 (35.8)	0.001	20 (4.6)	270 (61.5)	149 (33.9)	0.95
Parents	29 (1.4)	1,272 (62.8)	726 (35.8)		51 (2.9)	1,143 (65.9)	541 (31.2)	
Family	2 (2.3)	49 (55.7)	37 (42.0)		2 (4.2)	30 (62.5)	16 (33.3)	
Couple	3 (13.0)	16 (69.6)	4 (17.4)		2 (14.3)	6 (42.9)	6 (42.9)	
Other	1 (5.9)	10 (58.8)	6 (35.3)		0 (0.0)	0 (0.0)	1 (100)	
**Academic Program**								
Nursing	3 (1.0)	205 (69.3)	88 (29.7)	0.001	3 (1.0)	205 (69.3)	88 (29.7)	0.005
Medicine	10 (1.8)	392 (68.9)	167 (29.3)		10 (1.8)	392 (68.9)	167 (29.3)	
Physical Therapy	8 (2.7)	213 (70.8)	80 (26.6)		8 (2.7)	213 (70.8)	80 (26.6)	
Clinical Laboratory	0 (0.0)	205 (79.8)	52 (20.2)		0 (0.0)	205 (79.8)	52 (20.2)	
Dentistry	13 (2.6)	378 (75.1)	112 (22.3)		13 (2.6)	378 (75.1)	112 (22.3)	
Clinical Psychology	3 (1.0)	234 (75.2)	74 (23.8)		3 (1.0)	234 (75.2)	74 (23.8)	
**Level**								
First	2 (0.4)	290 (59.1)	199 (40.5)	0.55	7 (2.3)	191 (61.4)	113 (36.3)	0.003
Second	3 (1.4)	132 (61.4)	80 (37.2)		10 (4.5)	145 (65.9)	65 (29.5)	
Third	2 (0.7)	175 (62.7)	102 (36.6)		5 (2.3)	147 (66.2)	70 (31.5)	
Fourth	8 (2.3)	226 (64.2)	118 (33.5)		8 (2.8)	186 (64.8)	93 (32.4)	
Fifth	3 (1.1)	164 (62.6)	95 (36.3)		7 (2.1)	210 (63.6)	113 (34.2)	
Sixth	8 (3.0)	169 (62.4)	94 (34.7)		10 (3.6)	161 (58.3)	105 (38.0)	
Seventh	4 (3.2)	77 (61.1)	45 (35.7)		6 (2.6)	165 (70.8)	62 (26.6)	
Eighth	5 (3.4)	108 (72.5)	36 (24.2)		5 (3.2)	102 (66.2)	47 (30.5)	
Ninth	1 (2.8)	21 (58.3)	14 (38.9)		10 (8.5)	83 (70.3)	25 (21.2)	
Tenth	2 (4.8)	27 (64.3)	13 (31)		6 (8.3)	48 (66.7)	18 (25.0)	
Internship Rotation	0 (0.0)	7 (53.8)	6 (46.2)		1 (7.1)	11 (78.6)	2 (14.3)	
**Grade Point Average**								
Excellent (9-10)	9 (2.3)	229 (58.9)	151 (38.8)	0.445	11 (5.0)	126 (57.0)	84 (38.0)	0.003
Very Good (8-8.9)	19 (1.7)	735 (63.9)	396 (34.4)		28 (2.4)	779 (66.5)	365 (31.1)	
Good (7-7.9)	10 (1.6)	390 (62.4)	225 (36.0)		27 (3.9)	452 (65.1)	215 (31.0)	
Fail (<7)	0 (0.0)	42 (58.3)	30 (41.7)		9 (6.0)	92 (61.3)	49 (32.7)	

f_i, absolute frequency; %, percentage; M. mean; SD. standard deviation; p-value (statistical significance).
