# Supplementary material for: Lifestyles and academic stress among health sciences students at the National University of Chimborazo, Ecuador: a longitudinal study
Source: Front Public Health. 2024 Aug 12;12:1447649. doi: 10.3389/fpubh.2024.1447649 (PMC11345227; doi:10.3389/fpubh.2024.1447649)
Supplement: Supplementary file 2 [file Table_2.pdf]

Supplementary material 2: *Academic Stress Association X^2 (n= 2237) T1 y T2.*

Variables	First Moment (T1) Lightweight fi. (%)	Moderate fi. (%)	Severe fi. (%)	p T1	Second Moment (T2) Lightweight fi. (%)	Moderate fi. (%)	Severe fi. (%)	p T2
**Sex**								
Man	14 (2.2)	443 (68.6)	189 (29.3)	0.001	31 (4.8)	432 (66.9)	183 (28.3)	0.007
Woman	24 (1.5)	953 (59.9)	613 (38.6)		44 (2.8)	530 (33.3)	1017 (63.9)	
**Age**								
18 - 24	32 (1.5)	1,279 (61.6)	764 (36.8)	0.001	57 (3.1)	1,166 (64.0)	600 (32.9)	0.001
25 - 31	5 (3.3)	112 (73.7)	35 (23.09		16 (4.0)	275 (69.3)	106 (26.7)	
32 - 38	1 (14.3)	3 (42.9)	3 (42.9)		0 (0.0)	7 (53.8)	6 (46.2)	
39+	0 (0.0)	2 (100.0)	0 (0.0)		2 (50.0)	1 (25.0)	1 (25.0)	
**Marital status**								
Single	32 (1.5)	1,358 (62.3)	789 (36.2)	0.001	73 (3.4)	1,400 (64.7)	692 (32)	0.984
Married	3 (9.7)	22 (71.0)	6 (19.4)		2 (3.8)	35 (67.3)	15 (28.8)	
Divorced	0 (0.0)	1 (33.3)	2 (66.7)		0 (0.0)	5 (71.4)	2 (28.6)	
Cohabiting	3 (13.0)	15 (65.2)	5 (21.7)		0 (0.0)	9 (69.2)	4 (30.8)	
**Financial Dependence**								
Not applicable	3 (3.7)	49 (60.5)	29 (35.8)	0.001	20 (4.6)	270 (61.5)	149 (33.9)	0.95
Parents	29 (1.4)	1,272 (62.8)	726 (35.8)		51 (2.9)	1,143 (65.9)	541 (31.2)	
Family	2 (2.3)	49 (55.7)	37 (42.0)		2 (4.2)	30 (62.5)	16 (33.3)	
Couple	3 (13.0)	16 (69.6)	4 (17.4)		2 (14.3)	6 (42.9)	6 (42.9)	
Other	1 (5.9)	10 (58.8)	6 (35.3)		0 (0.0)	0 (0.0)	1 (100)	
**Academic Program**								
Nursing	3 (1.0)	191 (64.5)	102 (34.5)	0.005	16 (5.4)	189 (63.9)	91 (30.7)	0.003
Medicine	12 (2.1)	361 (63.4)	196 (34.4)		15 (2.6)	367 (64.5)	187 (32.9)	
Physical Therapy	8 (2.7)	212 (70.4)	81 (26.9)		8 (2.7)	215 (71.4)	78 (25.9)	
Clinical Laboratory	1 (0.4)	161 (62.6)	95 (37.0)		8 (3.1)	177 (68.9)	72 (28.0)	
Dentistry	7 (1.4)	286 (57.0)	209 (41.6)		17 (3.4)	289 (57.5)	197 (39.2)	
Clinical Psychology	7 (2.3)	185 (59.5)	119 (38.3)		11 (3.5)	212 (68.2)	88 (28.3)	
**Level**								
First	2 (0.4)	290 (59.1)	199 (40.5)	0.55	7 (2.3)	191 (61.4)	113 (36.3)	0.003
Second	3 (1.4)	132 (61.4)	80 (37.2)		10 (4.5)	145 (65.9)	65 (29.5)	
Third	2 (0.7)	175 (62.7)	102 (36.6)		5 (2.3)	147 (66.2)	70 (31.5)	
Fourth	8 (2.3)	226 (64.2)	118 (33.5)		8 (2.8)	186 (64.8)	93 (32.4)	
Fifth	3 (1.1)	164 (62.6)	95 (36.3)		7 (2.1)	210 (63.6)	113 (34.2)	
Sixth	8 (3.0)	169 (62.4)	94 (34.7)		10 (3.6)	161 (58.3)	105 (38.0)	
Seventh	4 (3.2)	77 (61.1)	45 (35.7)		6 (2.6)	165 (70.8)	62 (26.6)	
Eighth	5 (3.4)	108 (72.5)	36 (24.2)		5 (3.2)	102 (66.2)	47 (30.5)	
Ninth	1 (2.8)	21 (58.3)	14 (38.9)		10 (8.5)	83 (70.3)	25 (21.2)	
Tenth	2 (4.8)	27 (64.3)	13 (31)		6 (8.3)	48 (66.7)	18 (25.0)	
Internship Rotation	0 (0.0)	7 (53.8)	6 (46.2)		1 (7.1)	11 (78.6)	2 (14.3)	
**Grade Point Average**								
Excellent (9-10)	9 (2.3)	229 (58.9)	151 (38.8)	0.445	11 (5.0)	126 (57.0)	84 (38.0)	0.023
Very Good (8-8.9)	19 (1.7)	735 (63.9)	396 (34.4)		28 (2.4)	779 (66.5)	365 (31.1)	
Good (7-7.9)	10 (1.6)	390 (62.4)	225 (36.0)		27 (3.9)	452 (65.1)	215 (31.0)	
Fail (<7)	0 (0.0)	42 (58.3)	30 (41.7)		9 (6.0)	92 (61.3)	49 (32.7)	

f_i, absolute frequency; %, percentage; M, mean; SD, standard deviation; p-value, (statistical significance).
