# Supplementary material for: Lifestyles and academic stress among health sciences students at the National University of Chimborazo, Ecuador: a longitudinal study
Source: Front Public Health. 2024 Aug 12;12:1447649. doi: 10.3389/fpubh.2024.1447649 (PMC11345227; doi:10.3389/fpubh.2024.1447649)
Supplement: Supplementary file 3 [file Table_3.pdf]

frontiers

Supplementary material 3: *Perceived stress association X^2 (n= 2237) T1 y T2.*

	First Moment (T1)						Second Moment (T2)					
**Variables**	**Never**	**Almost never**	**Occasionally**	**Often**	**Very often**	**p T1**	**Never**	**Almost never**	**Occasionally**	**Often**	**Very often**	**p T2**
	**fi. (%)**	**fi. (%)**	**fi. (%)**	**fi. (%)**	**fi. (%)**		**fi. (%)**	**fi. (%)**	**fi. (%)**	**fi. (%)**	**fi. (%)**	
**Sex**												
Man	32 (5.0)	111 (17.2)	327 (50.6)	134 (20.7)	42 (6.5)	0.001	37 (5.7)	186 (28.8)	284 (44)	102 (15.8)	37 (5.7)	0.001
Woman	44 (2.8)	214 (13.5)	711 (44.7)	431 (27)	191 (12)		42 (2.6)	314 (19.7)	755 (47.5)	355 (22.3)	125 (7.9)	
**Age**												
18 - 24	69 (3.3)	292 (14.1)	958 (46.1)	534 (25.7)	223 (10.7)	0.165	57 (3.1)	386 (21.2)	860 (47.2)	395 (21.7)	125 (6.9)	0.001
25 - 31	7 (4.6)	33 (21.7)	73 (48)	29 (19.1)	10 (6.6)		21 (5.3)	111 (28.0)	170 (42.8)	62 (15.6)	33 (8.3)	
32 - 38	0 (0.0)	0 (0.0)	5 (71.4)	2 (28.6)	0 (0.0)		0 (0.0)	2 (15.4)	8 (61.5)	0 (0.0)	3 (23.1)	
39+	0 (0.0)	0 (0.0)	2 (100)	0 (0.0)	0 (0.0)		1 (25.0)	1 (25.0)	1 (25.0)	0 (0.0)	1 (25.0)	
**Level**												
First	10 (2.0)	49 (10.0)	216 (44.0)	150 (30.5)	66 (13.4)	0.001	7 (2.3)	48 (15.4)	151 (48.6)	80 (25.7)	25 (8.0)	0.001
Second	3 (1.4)	24 (11.2)	101 (47.0)	63 (29.3)	24 (11.2)		10 (4.5)	40 (18.2)	109 (49.5)	45 (20.5)	16 (7.3)	
Third	7 (2.5)	38 (13.6)	126 (45.2)	76 (27.2)	32 (11.5)		6 (2.7)	53 (23.9)	103 (46.4)	50 (22.5)	10 (4.5)	
Fourth	14 (4.0)	56 (15.9)	162 (46.0)	94 (26.7)	26 (7.4)		9 (3.1)	68 (23.7)	128 (44.6)	58 (20.2)	24 (8.4)	
Fifth	8 (3.1)	41 (15.6)	140 (53.4)	42 (16.0)	31 (11.8)		8 (2.4)	61 (18.5)	167 (50.6)	68 (20.6)	26 (7.9)	
Sixth	14 (5.2)	46 (17.0)	125 (46.1)	59 (21.8)	27 (10.0)		7 (2.5)	62 (22.5)	117 (42.4)	67 (24.3)	23 (8.3)	
Seventh	9 (7.1)	25 (19.8)	52 (41.3)	32 (25.4)	8 (6.3)		7 (3.0)	63 (27.0)	106 (45.5)	39 (16.7)	18 (7.7)	
Eighth	8 (5.4)	31 (20.8)	76 (51.0)	24 (16.1)	10 (6.7)		7 (4.5)	49 (31.8)	63 (40.9)	27 (17.5)	8 (5.2)	
Ninth	1 (2.7)	6 (16.2)	14 (37.8)	10 (27.0)	6 (16.2)		11 (9.3)	32 (27.1)	54 (45.8)	13 (11.0)	8 (6.8)	
Tenth	2 (4.8)	7 (16.7)	20 (47.6)	10 (23.8)	3 (7.1)		7 (9.7)	19 (26.4)	34 (47.2)	8 (11.1)	4 (5.6)	
Internship Rotation	0 (0.0)	2 (15.4)	6 (46.2)	5 (38.5)	0 (0.0)		0 (0.0)	5 (35.7)	7 (50.0)	2(14.3)	0 (0.0)	
**Financial Dependence**												
Not applicable	3 (3.7)	20 (24.7)	31 (38.3)	20 (24.7)	7 (8.6)	0.03	18 (4.1)	99 (22.6)	117 (40.3)	108 (24.6)	37 (8.4)	0.88
Parents	65 (3.2)	287 (14.2)	948 (46.7)	519 (25.6)	209 (10.3)		56 (3.2)	391 (22.5)	832 (48)	338 (19.5)	118 (6.8)	
Family	3 (3.4)	11 (12.5)	39 (44.3)	23 (26.1)	12 (13.6)		3 (6.3)	8 (16.7)	24 (50)	8 (16.7)	5 (10.4)	
Couple	2 (8.7)	5 (21.7)	11 (47.8)	2 (8.7)	3 (13.0)		2 (14.3)	2 (14.3)	6 (42.9)	2 (14.3)	2 (14.3)	
Other	3 (17.6)	2 (11.8)	9 (52.9)	1 (5.9)	2 (11.8)		0 (0.0)	0 (0.0)	0 (0.0)	1 (100)	0 (0.0)	
**Economic activity**												
Yes	2 (4.1)	13 (26.5)	18 (36.7)	11 (22.4)	5 (10.2)	0.395	16 (3.9)	95 (23.3)	161 (39.5)	100 (24.5)	36 (8.8)	0.022
No	74 (3.38)	312 (14.2)	1020 (46.61)	554 (25.3)	228 (10.4)		63 (3.4)	405 (22.1)	878 (48.0)	357 (19.5)	126 (6.9)	

f_i, absolute frequency; %, percentage; M, mean; SD, standard deviation; p-value, (statistical significance).
