# Supplementary material for: Lifestyles and academic stress among health sciences students at the National University of Chimborazo, Ecuador: a longitudinal study
Source: Front Public Health. 2024 Aug 12;12:1447649. doi: 10.3389/fpubh.2024.1447649 (PMC11345227; doi:10.3389/fpubh.2024.1447649)
Supplement: Supplementary file 4 [file Table_4.pdf]

Supplementary material 4: *Perceived coping association X^2 (n= 2237) T1 y T2.*

Variables	First Moment (T1)						Second Moment (T2)					
	Never	Almost never	Occasionally	Often	Very often	p T1	Never	Almost never	Occasionally	Often	Very often	p T2
	fi. (%)	fi. (%)	fi. (%)	fi. (%)	fi. (%)		fi. (%)	fi. (%)	fi. (%)	fi. (%)	fi. (%)	
**Sex**												
Man	14 (2.2)	110 (17)	320 (49.5)	160 (24.8)	42 (6.5)	0.415	29 (4.5)	141 (21.8)	306 (47.4)	122 (18.9)	48 (7.4)	0.002
Woman	49 (3.1)	233 (14.6)	831 (52.2)	378 (23.8)	100 (6.3)		51 (3.2)	271 (17.0)	827 (52.0)	360 (22.6)	82 (5.2)	
**Age**												
18 - 24	59 (2.8)	308 (14.8)	1075 (51.8)	503 (24.2)	131 (6.3)	0.14	60 (3.3)	314 (17.2)	940 (51.6)	407 (22.3)	102 (5.6)	0.03
25 - 31	4 (2.6)	35 (23.0)	72 (47.4)	30 (19.7)	11 (7.2)		19 (4.8)	93 (23.4)	185 (46.6)	73 (18.4)	27 (6.8)	
32 - 38	0 (0.0)	0 (0.0)	4 (57.1)	3 (42.0)	0 (0.0)		0 (0.0)	4 (30.8)	7 (53.8)	1 (7.7)	1 (7.7)	
39+	0 (0.0)	0 (0.0)	0 (0.0)	2 (100)	0 (0.0)		1 (25.0)	1 (25.0)	1 (25.0)	1 (25.0)	0 (0.0)	
**Marital status**												
Single	56 (2.6)	332 (15.2)	1126 (51.7)	527 (24.2)	139 (6.4)	0.001	78 (3.6)	394 (18.2)	1,098 (50.7)	468 (21.6)	127 (5.9)	0.695
Married	3 (9.7)	5 (16.1)	14 (45.2)	8 (25.8)	1 (3.2)		1 (1.9)	12 (23.1)	25 (48.1)	11 (21.2)	3 (5.8)	
Divorced	1 (33.3)	1 (33.3)	0 (0.0)	1 (33.3)	0 (0.0)		1 (14.3)	3 (42.9)	3 (42.9)	0 (0.0)	0 (0.0)	
Cohabiting	3 (13.0)	5 (21.7)	11 (47.8)	2 (8.7)	2 (8.7)		0 (0.0)	3 (23.1)	7 (53.8)	3 (23.1)	0 (0.0)	
**Financial Dependence**												
Not applicable	2 (2.5)	19 (23.5)	39 (48.1)	15 (18.5)	6 (7.4)	0.134	18 (4.1)	87 (19.8)	214 (48.7)	91 (20.7)	29 (6.6)	0.658
Parents	54 (2.7)	305 (15)	1052 (51.9)	487 (24)	130 (6.4)		57 (3.3)	311 (17.9)	892 (51.4)	379 (21.8)	96 (5.5)	
Family	3 (3.4)	13 (14.8)	38 (43.2)	28 (31.8)	6 (6.8)		4 (8.3)	9 (18.8)	22 (45.8)	9 (18.8)	4 (8.3)	
Couple	3 (13)	2 (8.7)	13 (56.5)	5 (21.7)	0 (0.0)		1 (7.1)	4 (28.6)	5 (35.7)	3 (21.4)	1 (7.1)	
Other	1 (5.9)	4 (23.5)	9 (52.9)	3 (17.6)	0 (0.0)		0 (0.0)	1 (100)	0 (0.0)	0 (0.0)	0 (0.0)	
**Academic Program**												
Nursing	7 (2.4)	48 (16.2)	155 (52.4)	63 (21.3)	23 (7.8)	0.056	14 (4.7)	49 (16.6)	160 (54.1)	58 (19.6)	15 (5.1)	0.184
Medicine	20 (3.5)	94 (16.5)	277 (48.7)	142 (25)	36 (6.3)		21 (3.7)	106 (18.6)	298 (52.4)	114 (20)	30 (5.3)	
Physical Therapy	10 (3.3)	45 (15)	159 (52.8)	62 (20.6)	25 (8.3)		11 (3.7)	54 (17.9)	162 (53.8)	58 (19.3)	16 (5.3)	
Clinical Laboratory	9 (3.5)	28 (10.9)	136 (52.9)	77 (30)	7 (2.7)		1 (0.4)	47 (18.3)	123 (47.9)	67 (26.1)	19 (7.4)	
Dentistry	7 (1.4)	85 (16.9)	273 (54.3)	110 (21.9)	28 (5.6)		22 (4.4)	98 (19.5)	250 (49.7)	101 (20.1)	32 (6.4)	
Clinical Psychology	10 (3.2)	43 (13.8)	151 (48.6)	84 (27)	23 (7.4)		11 (3.5)	58 (18.6)	140 (45)	84 (27)	18 (5.8)	
**Level**												
First	12 (2.4)	57 (11.6)	253 (51.5)	132 (26.9)	37 (7.5)	0.031	9 (2.9)	46 (14.8)	166 (53.4)	73 (23.5)	17 (5.5)	0.137
Second	5 (2.3)	29 (13.5)	124 (57.7)	50 (23.3)	7 (3.3)		8 (3.6)	33 (15)	116 (52.7)	47 (21.4)	16 (7.3)	
Third	5 (1.8)	43 (15.4)	134 (48.0)	76 (27.2)	21 (7.5)		8 (3.6)	43 (19.4)	112 (50.5)	51 (23.0)	8 (3.6)	
Fourth	11 (3.1)	63 (17.9)	169 (48.0)	93 (26.4)	16 (4.5)		10 (3.5)	51 (17.8)	144 (50.2)	62 (21.6)	20 (7.0)	

Fifth	4 (1.5)	40 (15.3)	135 (51.5)	57 (21.8)	26 (9.9)		8 (2.4)	59 (17.9)	171 (51.8)	73 (22.1)	19 (5.8)	
Sixth	11 (4.1)	47 (17.3)	143 (52.8)	58 (21.4)	12 (4.4)		8 (2.9)	51 (18.5)	138 (50)	65 (23.6)	14 (5.1)	
Seventh	5 (4.0)	20 (15.9)	64 (50.8)	27 (21.4)	10 (7.9)		4 (1.7)	48 (20.6)	118 (50.6)	52 (22.3)	11 (4.7)	
Eighth	8 (5.4)	29 (19.5)	82 (55.0)	26 (17.4)	4 (2.7)		7 (4.5)	39 (25.3)	72 (46.8)	28 (18.2)	8 (5.2)	
Ninth	0 (0.0)	5 (13.5)	18 (48.6)	9 (24.3)	5 (13.5)		11 (9.3)	23 (19.5)	55 (46.6)	18 (15.3)	11 (9.3)	
Tenth	2 (4.8)	9 (21.4)	22 (52.4)	5 (11.9)	4 (9.5)		7 (9.7)	16 (22.2)	32 (44.4)	11 (15.3)	6 (8.3)	
Internship Rotation	0 (0.0)	1 (7.7)	7 (53.8)	5 (38.5)	0 (0.0)		0 (0.0)	3 (21.4)	9 (64.3)	2 (14.3)	0 (0.0)	
**Grade Point Average**												
Excellent (9-10)	21 (5.4)	59 (15.2)	179 (46.0)	104 (26.7)	26 (6.7)		10 (4.5)	29 (13.1)	111 (50.2)	55 (24.9)	16 (7.2)	
Very Good (8-8.9)	27 (2.3)	161 (14.0)	603 (52.4)	282 (24.5)	78 (6.8)	0.031	32 (2.7)	226 (19.3)	593 (50.6)	248 (21.2)	73 (6.2)	0.122
Good (7-7.9)	14 (2.2)	109 (17.4)	329 (52.6)	139 (22.2)	34 (5.4)		30 (4.3)	129 (18.6)	346 (49.9)	150 (21.6)	39 (5.6)	
Fail (<7)	1 (1.4)	14 (19.4)	40 (55.6)	13 (18.1)	4 (5.6)		8 (5.3)	28 (18.7)	83 (55.3)	29 (19.3)	2 (1.3)	

f_i, absolute frequency; %, percentage; M, mean; SD, standard deviation; p-value, (statistical significance).
